# Intermittent Hypoxic Exposure Reduces Endothelial Dysfunction

**DOI:** 10.1155/2020/6479630

**Published:** 2020-08-19

**Authors:** Agnieszka Zembron-Lacny, Anna Tylutka, Eryk Wacka, Edyta Wawrzyniak-Gramacka, Dariusz Hiczkiewicz, Anna Kasperska, Miłosz Czuba

**Affiliations:** ^1^Department of Applied and Clinical Physiology, Collegium Medicum University of Zielona Gora, Poland; ^2^Students' Scientific Society of Physiology and Pathology, Collegium Medicum University of Zielona Gora, Poland; ^3^Department of Physiology, University School of Physical Education Poznan, Poland

## Abstract

Intermittent exposure to hypoxia (IHE) increases the production of reactive oxygen and nitrogen species as well as erythropoietin (EPO), which stimulates the adaptation to intense physical activity. However, several studies suggest a protective effect of moderate hypoxia in cardiovascular disease (CVD) events. The effects of intense physical activity with IHE on oxi-inflammatory mediators and their interaction with conventional CVD risk factors were investigated. Blood samples were collected from elite athletes (control *n* = 6, IHE *n* = 6) during a 6-day IHE cycle using hypoxicator GO2 altitude. IHE was held once a day, at least 2 hours after training. In serum, hydrogen peroxide (H_2_O_2_), nitric oxide (NO), 3-nitrotyrosine (3-Nitro), proinflammatory cytokines (IL-1*β* and TNF*α*), high sensitivity C-reactive protein (hsCRP), and heat shock protein 27 (HSP27) were determined by the commercial immunoenzyme (ELISA kits) or colorimetric methods. Serum erythropoietin (EPO) level was measured by ELISA kit every day of hypoxia. IHE was found to significantly increase H_2_O_2_, NO, and HSP27 but to decrease 3NT concentrations. The changes in 3NT and HSP27 following hypoxia proved to enhance NO bioavailability and endothelial function. In the present study, the oxi-inflammatory mediators IL-1*β* and hsCRP increased in IHE group but they did not exceed the reference values. The serum EPO level increased on the 3^rd^ day of IHE, then decreased on 5^th^ day of IHE, and correlated with NO/H_2_O_2_ ratio (*r*_s_ = 0.640, *P* < 0.05). There were no changes in haematological markers contrary to lipoproteins such as low-density lipoprotein (LDL) and non-high-density lipoprotein (non-HDL) which showed a decreasing trend in response to hypoxic exposure. The study demonstrated that IHE combined with sports activity reduced a risk of endothelial dysfunction and atherogenesis in athletes even though the oxi-inflammatory processes were enhanced. Therefore, 6-day IHE seems to be a potential therapeutic and nonpharmacological method to reduce CVD risk, especially in elite athletes participating in strenuous training.

## 1. Introduction

Systemic exposure to hypoxia increases the production of reactive oxygen and nitrogen species as well as erythropoietin (EPO), which stimulates the adaptation to intense exercise [1]. The narrative review by Ploszczyca et al. [2] reported that blood EPO level increased within several hours and reached its peak after 1-3 days of acute hypoxic exposure. The high EPO level is known to result in improved erythropoiesis and exercise capacity. After returning to normoxia conditions, EPO returns to its baseline value immediately or declines below the initial value [3, 4]. Therefore, hypoxic exposure is one of the methods which have been used for decades by professional athletes to increase endurance, strength, and speed, to avoid fatigue and to improve recovery [5–7]. A few epidemiologic and physiologic observations have demonstrated positive effects of hypoxia conditions on cardiovascular health [8–10]. Being born at high altitude was shown to have an independent protective effect against coronary heart disease. Data from 4.2 million individuals demonstrated that mortality from cardiovascular disease (CVD) decreased linearly with increasing altitude [11]. Over recent years, intermittent hypoxic exposure (IHE) has been introduced into sport. IHE is a method by which athletes are exposed to short bouts of moderate hypoxia (9-12% O_2_), interspersed with periods of normal air. The available studies have reported substantial improvements in sea level endurance and anaerobic performance after IHE [12]. Beside the effects which hypoxia exerts on physical performance, there is more than one study which has shown that IHE might favourably affect vascular endothelial activity [13–16].

The hypoxic exposure has a potential impact on endothelium thereby reducing atherosclerotic risk factors, retarding arterial wall ageing, delaying development of endothelial dysfunction, and preserving vascular function. Furthermore, regular physical activity with hypoxia exposure increases NO production via iNOS and eNOS [17], and thus, it could modify the lipid profile and inhibit the production of proinflammatory cytokines: tumour necrosis factor *α* (TNF*α*) and interleukin 1*β* (IL-1*β*) [18–21]. The majority of such observations were performed in healthy nonactive subjects or patients with coronary artery disease [10, 22–25]. According to Angeli et al. [25], the generation of inflammatory mediators in physically active men causes disturbances to vascular endothelial activity which precedes the development of arteriosclerosis. Maron et al. [26] reported that 18.5% incidence of sudden death in young athletes (<35 years) were related to vascular endothelial dysfunction and atherosclerotic coronary artery disease. Agrotou et al. [27] demonstrated that the type of anaerobic exercise was an important determinant of subclinical atherosclerosis such as intima-media thickness and flow-mediated dilation. So far, little is known of the effects of intensive physical training in combination with hypoxic exposure on emerging markers of cardiovascular risk. The study is aimed at evaluating the oxi-inflammatory mediators and their interaction with conventional CVD risk factors after intermittent hypoxia exposure.

## 2. Material and Methods

### 2.1. Subjects

Twenty elite male wrestlers, members of the national team, were observed during preparatory periods for the new competition season (endurance training 53%, directed training 9% and special power training 38%). Each athlete underwent a thorough screening, including a full medical evaluation in National Centre for Sports Medicine. Exclusion criteria included serious orthopaedic injury (*n* = 3), medications (*n* = 2), dehydration (*n* = 2), and anaemia (*n* = 1) identified during the observation. Eventually, twelve athletes were included in the study (Table 1). The first day of the training camp, athletes were randomly assigned to a control group (*n* = 6) and a hypoxia group IHE (*n* = 6) in a single blind fashion. Both groups performed the same IHE program whereby the control group was supplied with oxygen equivalent to ambient air (20.9% O_2_). Throughout the camp all athletes lived at the same accommodation and followed the same training schedule, sleeping time, and diet. Daily energy value of food offered on the menu did not exceed 5200 kcal, and the protein dose varied from 1.6 to 1.8 g/kg of body mass. During the camp, the wrestlers consumed an isotonic sports drink Vitargo (osmolality 317 mOsm/kg H_2_O) or plain water. Athletes did not take any nutrition supplements that could interfere with oxi-inflammatory evaluation. All the subjects were informed of the aim of the study and signed a written consent to participate in the project. The protocol of the study was approved by the ethics committee at Medical University Poznan (No. 550/11), in accordance with the Helsinki Declaration.

### 2.2. Body Composition

Body mass (BM) and body composition (fat-free mass FFM and fat mass FM) were estimated using Tanita Body Composition Analyser MC-418 (Japan) calibrated prior to each test session in accordance with the manufacturer's guidelines. Duplicate measures were taken with the participant in a standing position; the average value was used for the final analysis. The recurrence of measurement amounted to 98%. The measurements were taken between 7.00 and 8.00 a.m. before blood sampling.

### 2.3. Intermittent Hypoxic Exposure

The passive 6-day intermittent hypoxic exposure (IHE) was conducted under medical supervision according to the procedure by Hinickson et al. [12] using the GO_2_ altitude hypoxicator (Australia) at the Olympic Sports Centre. The hypoxicator was able to simulate the height of 2,500 to 6,500 metres above sea level by regulating oxygen levels at FiO_2_ of 14-9% (fraction of inspired oxygen). IHE parameters were determined after the preliminary assessment of athlete's ability to adapt to hypoxic gas mixtures. The hypoxic test included the measurements of the time for the blood saturation to drop to 85% with FiO_2_ = 12% (equivalent to 4,500 m above sea level) and the time for the blood saturation to return to 95% in normoxia. Based on the hypoxic test results, the IHE protocol was determined for each athlete. IHE was applied once a day, at least 2 hours after sports training. Each IHE session consisted of 6 doses of 3-8-minute periods of hypoxia (at FiO_2_ of 14-12%) interrupted by 3-5-minute periods of normoxia and repeated for 60-80 minutes. Hypoxic exposure started with two sessions of acclimatisation with FiO_2_ = 13.5% (equivalent to 3,000 m above sea level), and then reduced to FiO_2_ = 12% (equivalent to 4,500 m above sea level). The blood saturation (SpO_2_) and heart rate (HR) were individually monitored during every IHE session. SpO_2_ oscillated from 90.8 ± 2.4% on 1^st^ day to 91.4 ± 5.6% on 6^th^ day whereas HR oscillated from 76.0 ± 7.8 bpm on 1^st^ day to 76.1 ± 11.8 bpm on 6^th^ day in IHE group.

### 2.4. Pro- and Anti-inflammatory Mediators

Serum TNF*α* and IL-1*β* levels were determined by R&D Systems kits (USA), and their detection limits were 0.038 pg/mL and 0.023 pg/mL, respectively. Oxidized low-density lipoprotein (ox-LDL) and high-sensitivity C-reactive protein (hsCRP) concentrations were determined using commercial kits from EIAab Science kit (China) and DRG International (USA). The detection limits for ox-LDL and hsCRP amounted to 0.312 ng/mL and 0.001 mg/L, respectively. Hydrogen peroxide (H_2_O_2_), nitric oxide (NO), and the marker of NO bioavailability (3-nitrotyrosine, 3NT) concentrations were determined by colorimetric and enzyme immunoassay methods using the Oxis Research kits (USA). H_2_O_2_, NO, and 3NT detection limits reached the levels of 6.25 *μ*mol/L, 0.5 *μ*mol/L, and 2 nmol/L, respectively. Heat shock protein 27 (HSP27) was measured with a Calbiochem kit (USA) with the detection limit at 0.2 ng/mL. All samples were analysed in duplicate or triplicate in a single assay to avoid interassay variability. The average intra-assay coefficient of variation for the kits was set at <5-8%.

### 2.5. Lipoprotein-Lipid Profile and Haematological Variables

Total cholesterol (TC), high-density lipoproteins (HDL), and low-density lipoproteins (LDL) as well as triglycerides (TG) were determined by professional laboratory company Diagnostyka (Poland, ISO 15189). The non-HDL cholesterol was calculated by subtracting HDL from the total cholesterol concentration. The haematological markers, i.e., haemoglobin (HB), red blood cells (RBC), reticulocytes (RET), haematocrit (HTC), mean corpuscular volume (MCV), mean corpuscular haemoglobin (MCH), mean corpuscular concentration (MCHC), and red blood cell distribution (RDW) were also determined by Diagnostyka (Poland, ISO 15189). Erythropoietin (EPO) was determined by enzyme immunoassay methods using the R&D Systems kits (USA). The detection limits for EPO was set at 0.6 mlU/mL.

### 2.6. Statistical Analysis

Statistical analyses were performed by means of statistical software Statistica 13.1 (StatSoft Inc., Tulsa, OK, USA). The assumptions for the use of parametric or nonparametric tests were checked using the Shapiro-Wilk test to evaluate the normality of the distributions. The significant differences in mean values between the groups (control vs. IHE) were assessed by the one-way ANOVA. If the normality was violated, the Mann-Whitney nonparametric test was used. The comparisons of repeated measurements (1^st^ day vs. 6^th^ day of sports training or hypoxic exposure) were assessed by the Wilcoxon signed-ranks test. The associations between the measured parameters were analysed using Spearman's rank correlation (*r*_s_, Spearman rank correlation coefficient). Statistical significance was set at *P* < 0.05. The results are expressed as mean, standard errors of the means and median (*x* ± SEM, Me).

## 3. Results

### 3.1. Pro- and Anti-inflammatory Mediators (Table 2)

The concentrations of TNF*α*, IL-1*β*, H_2_O_2_, NO, and HSP27 did not change in control group, except for 3-nitrotyrosine which increased by approximately 20%. In the control group, 3NT moderately correlated with conventional CVD risk factors, such as TC (*r*_s_ = 0.439, *P* < 0.05), and non-HDL (*r*_s_ = 0.418, *P* < 0.05). The oxi-inflammatory mediators IL-1*β*, TNF*α*, and hsCRP were found to increase in the IHE group. NO concentration and its bioavailability, demonstrated by low level of 3NT, were particularly enhanced by 6-day IHE. NO concentration increased by approximately 85% in IHE group and inversely correlated with 3NT (*r*_s_ = −0.601, *P* < 0.05). HSP27 significantly increased in IHE group and also correlated with NO (*r*_s_ = 0.741, *P* < 0.05).

### 3.2. Lipoprotein-Lipid Profile (Table 3)

TG and HDL concentrations were found to be at similar levels in all subjects. High levels of TC > 200 mg/dL and LDL > 130 mg/dL were observed in 33% and 25% of the subjects, respectively. Finally, non-HDL exceeded the level of 145 mg/dL in 33% of the athletes. Hypoxic exposure induced significant 24% decrease in TC, 36% decrease in LDL, and 28% decrease in non-HDL on the 6^th^ day compared to controls. ox-LDL concentration was observed to decrease in both groups on the 6^th^ day, but it was significantly lower in IHE than in controls.

### 3.3. Haematological Variables (Table 4)

**Figure 1:**
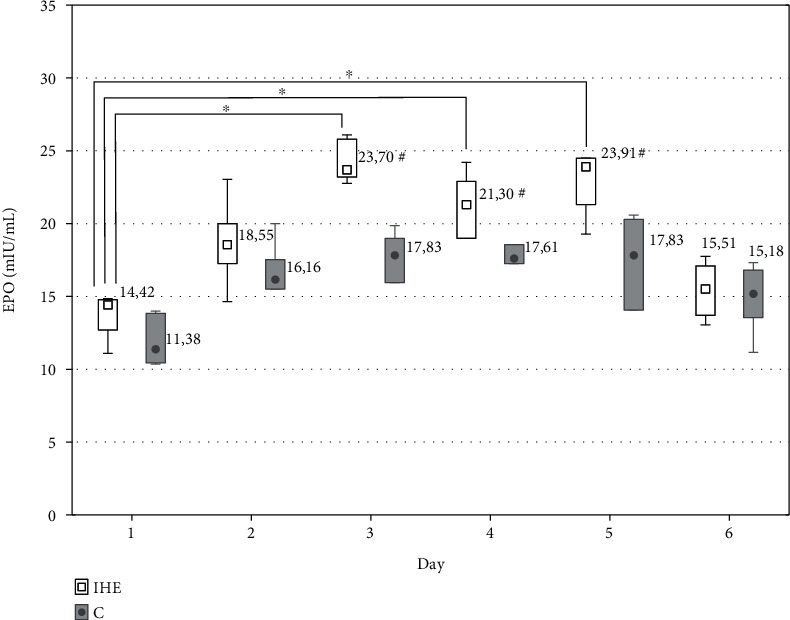
Median values in serum EPO concentration during the intermittent hypoxic exposure protocol in the experimental (IHE) and control group (C); ^∗^*P* < 0.05 statistically significant differences in relation to the initial measurements; ^#^*P* < 0.05 statistically significant differences in between groups.

There were no changes in haematological markers RET, RBC, HB, and HTC during the 6-day observation. The serum EPO level significantly increased from 13.71 ± 1.53 mlU/mL on the 1^st^ day to 24.20 ± 1.40 mlU/mL on the 3^rd^ day (approximately 1.5-fold compared to the control group), and then decreased on the 5^th^ day (15.42 ± 1.84 mlU/mL) during hypoxic exposure (Figure 1). The differences between the controls and IHE group were statistically significant (*P* < 0.05). The concentration of EPO highly correlated with NO/H_2_O_2_ ratio (*r*_s_ = 0.640, *P* < 0.05), which indicates a substantial share of NO and H_2_O_2_ in erythropoietin synthesis.

## 4. Discussion

Low-grade systemic inflammation may be involved in vascular inflammation and in pathogenesis of several chronic pathological conditions. Recent findings confirm that physical activity induces an increase in the systemic levels of a number of cytokines and chemokines with pro- and anti-inflammatory properties [28–33]. It contributes to the progression of atherosclerosis and increases the risk of CVD events. Proinflammatory cytokines play a pivotal role in endothelial dysfunction and IL-1*β* and TNF*α* belong to the ones that have attracted the most attention because of their crucial involvement in CVD. IL-1*β* and TNF*α* are mainly secreted by monocytes, macrophages, and neutrophils. These proinflammatory cytokines can affect immune cells such as macrophages, dendritic cells, and neutrophils as well as nonimmune cell types, including vascular endothelial and smooth muscle cells, to induce inflammatory response and other atherogenic effects [21, 25, 34, 35]. Direct evidence of TNF*α*-stimulated vascular dysfunction was provided by a study of intra-arterial TNF*α* administration in humans. In healthy volunteers, the administration of a lower TNF*α* dose (17 ng/min) for 60 minutes was observed to induce an increase in basal vascular resistance, which was blocked by pre-treatment with a NO synthase inhibitor. The authors concluded that the observed effects of TNF*α* were likely to be mediated by the reduced bioavailability of NO [21]. In our previous studies, we observed a strong relation of 3NT with TNF*α* in athletes after 14 days of strenuous sports training [33]. In the present study, the athletes from the control group did not demonstrate elevated levels of IL-1*β*, TNF*α* and hsCRP. However, hsCRP was found to be elevated after intermittent hypoxia although it did not exceed the reference range of 0-5 mg/L.

However, NO concentration and its bioavailability significantly increased by 85% after IHE, which can improve endothelial function. Many studies have demonstrated that intermittent hypoxia increases NO production in a variety of model systems, including cells, blood vessels, muscles, and isolated hearts [8, 9, 36–40]. Inducible nitric oxide synthase (iNOS) appears to be the primary source of cardioprotective NO during hypoxic exposure [41, 42]. Chronic hypoxia was reported to enhance myocardial iNOS expression [43–45], while an inhibition of iNOS was shown to abolish the associated cardioprotection [41]. Some reports suggested that endothelial NOS expression was similarly elevated in hypoxia-exposed animals [37, 38, 45]. Moreover, a direct reduction of nitrite to NO was also observed to be enhanced by intermittent hypoxia [8, 45].

NO and H_2_O_2_ are involved in signal transduction pathways as part of the O_2_-sensing mechanism stabilising transcription factor HIF-1 and regulating erythropoietin expression. EPO regulates the process of haematopoiesis, stabilises vascular integrity, increases the number of endothelial cells, and protects these cells against ischemia and apoptosis. In our study, the serum EPO level significantly increased on the 3^rd^ day and then decreased on the 5^th^ day during hypoxic exposure (Figure 1). The increase in EPO concentration in response to hypoxia depends upon the duration and severity of hypoxia [46], with noticeable increases in the circulating EPO after short-term exposures to hypoxia (altitude ~4,000 m for ~84 min) [47], reaching peak levels after ~48 h [48]. Nocturnal hypoxia (12-14 h/night) has also been shown to stimulate EPO secretion but the elevation of its concentration is not sustained [49]. EPO is a major area of research regarding its protective effect against ischaemia in the heart. However, whether intermittent hypoxia induces an increase in EPO in patients with CVD is yet to be clarified [50]. According to Heeschen et al. [51], EPO is a potent physiologic stimulus for endothelial progenitor cell mobilisation. The EPO expression during intermittent hypoxia is more dependent on changes in H_2_O_2_, whereas in nonhypoxic conditions it is related to NO, pro-inflammatory cytokines IL-*β* and TNF*α*, and growth factors [52]. Interestingly, 6-day intermittent hypoxia was observed to elevate H_2_O_2_ and NO levels. Although EPO did not correlate with H_2_O_2_ and NO separately_,_ it highly correlated with the ratio of NO to H_2_O_2_. This means that EPO synthesis can require simultaneous changes in both H_2_O_2_ and NO levels. It has been described that HIF-1 transcriptional activity and EPO expression are achieved through two parallel pathways, i.e., a decrease in O_2_-dependent hydroxylation of HIF-1 and S-nitrosylation of the HIF-1 pathway components [7, 8]. Furthermore, in response to EPO, the expression of NOS is also elevated and it is followed by subsequent NO and cGMP production. Beleslin-Cokic et al. [53] provided evidence that intermittent hypoxia increased the capacity of endothelial cells to produce NO.

The mechanisms involved in the generation of reactive oxygen and nitrogen species are critical for endothelial function [8, 34, 35, 45]. NO and H_2_O_2_ overproduction as well as nitration of many proteins decrease enzyme activity, disrupt metabolism and cellular detoxification, perturb cytoskeletal organization, and ultimately contribute to the cytotoxic effects of peroxynitrite [54, 55]. 3NT measurement is required to confirm the presence of peroxynitrite in biological systems [55]. In our study, 3NT increased by approximately 20% in the control group and positively correlated with conventional CVD risk factors such as TC, LDL, and non-HDL. This confirms the suggestion of Tejero et al. [55] that detection and quantification of 3NT can be used as an indicator for the pathological processes in vascular endothelium. Importantly, in this study the nitration of tyrosine was observed to decrease by approximately 20% after intermittent hypoxia, which indicates an increase of NO bioavailability for vascular smooth muscle cells and cardiac myocytes. Meerson et al. [9] demonstrated that the moderate intermittent hypoxic conditioning mobilised mechanisms that defended myocardium from acute or prolonged ischemia and prevented life-threatening arrhythmias. According to Mallet et al. [8], intermittent hypoxic exposure increases cardiac resistance to ischemia-reperfusion stress and can be safely used in clinical practice to protect subjects with developing coronary disease or those awaiting cardiac procedures. In sport, IHE is commonly used to increase physical performance. However, our study demonstrated that IHE can simultaneously impact on endothelial function in athletes participating in sports which include strength elements.

In the last few years, the dose-response relationship of exercise and CVD outcomes and whether high volume of exercise may accelerate endothelial dysfunction and atherosclerosis have been a matter of debate [33, 34, 56, 57]. Prior observations showed a significantly higher coronary artery calcification in marathon runners compared with the control subjects who were matched for both age and CVD risk factors [58]. This contrasts with other studies that found either no association or an inverse relationship between physical activity or fitness and atherosclerosis [59, 60]. Identification of risk factors such as elevated LDL and non-HDL, hypertension, diabetes mellitus, and a family history of premature CVD is of crucial importance in athletes. High concentrations of blood cholesterol, LDL, and non-HDL in particular, in 33% of our athletes can be the major risk factors of atherosclerosis, especially after their sports career is completed. The strenuous 6-day sport training in our study led to an increase in non-HDL to the level of 163 ± 8 mg/dL whereas IHE reduced non-HDL to the level of 118 ± 36 mg/dL. Therefore, we have evidence that intermittent hypoxia exposure could aid the pharmacological lipid-lowering therapy.

A small heat shock protein, HSP27, is one of many molecules which play a protective role against endothelial dysfunction. It is involved in a wide variety of cellular processes such as apoptosis, oxi-inflammation, cell migration, and maintenance of arterial wall homeostasis [61]. HSP27 in atherosclerotic plaques diminishes with progression of the stage of the pathology and is thereby viewed as a potential biomarker [62–64]. The protective effect of HSP27 is associated with a decrease in reactive oxygen species during oxidative stress [65, 66]. In our study, the measurement of HSP27 level revealed a lowering tendency after 6-day strenuous sports training. A similar result was observed in patients with carotid artery disease and haemodialysis patients; however, the levels were shown to increase in acute coronary syndromes [62, 65]. In humans, low serum HSP27 levels were also demonstrated to be inversely associated with plaque burden in coronary artery and the prognosis of future adverse clinical events [65–68]. The introduction of intermittent hypoxia exposure to training schedule resulted in an increase in HSP27 concentration, which may prevent the development of atherosclerosis in athletes, especially after the end of their professional sports career.

## 5. Conclusions

In this study, we demonstrated for the first time that intermittent hypoxic exposure combined with physical activity may reduce a risk of endothelial dysfunction by an increase in nitric oxide bioavailability and circulating HSP27 level. Therefore, a 6-day intermittent hypoxic exposure provides potential therapeutic benefits, especially in athletes undergoing strenuous training schedule. However, the transfer of hypoxic exposure intervention to a clinical setting requires further studies in patients with cardiovascular diseases.

## 6. Limitations

The limitations of the study include a relatively small number of subjects, a short 6-day period of observation, and no direct assessment of endothelial function. Moreover, a limited number of available epidemiologic and physiologic observations in athletes make it difficult to explain the impact of IHE on endothelial function.

## Figures and Tables

**Table 1:** Anthropometrics and body composition of subjects (mean ± SEM).

Parameter	Control, *n* = 6	IHE, *n* = 6	Control vs. IHE, *P* value
Age (yrs.)	20.7 ± 0.8	21.0 ± 0.5	0.709
Height (cm)	183.5 ± 2.9	173.8 ± 1.8	0.240
Weight (kg)	77.6 ± 5.5	78.5 ± 2.4	0.906
BMI (kg/m^2^)	22.9 ± 1.0	25.9 ± 0.6	0.242
%FM	9.0 ± 1.3	10.0 ± 0.4	0.427
FM (kg)	7.2 ± 1.5	7.9 ± 0.5	0.677
%FFM	91.0 ± 1.3	89.8 ± 0.4	0.372
FFM (kg)	70.4 ± 4.5	70.6 ± 1.9	0.978

Abbreviations: BMI body mass index, FM fat mass, FFM fat-free mass, SEM standard error of the mean. The measurements in groups are compared by the one-way ANOVA or the Manna-Whitney non-parametric test (if the normality assumption is violated).

**Table 2:** Pro- and anti-inflammatory markers of atherosclerosis before and after 6-day intermittent hypoxic exposure (mean ± SEM, Me).

	Control				1^st^ day vs. 6^th^ day	IHE				1^st^ day vs. 6^th^ day	Control vs. IHE 6^th^ day, *P* value
	1^st^ day, *n* = 6		6^th^ day, *n* = 6			1^st^ day, *n* = 6		6^th^ day, *n* = 6			
	*x* ± SEM	Me	*x* ± SEM	Me		*x* ± SEM	Me	*x* ± SEM	Me		
TNF*α* (pg/mL)	3.82 ± 0.06	3.82	3.70 ± 0.07	3.71	>0.05	3.93 ± 0.04	3.93	4.03 ± 0.12	4.04	>0.05	>0.05
IL-1*β* (pg/mL)	2.06 ± 0.09	2.05	2.53 ± 0.13	2.44	>0.05	2.09 ± 0.10	2.03	2.68 ± 0.12	2.64	<0.05	>0.05
hsCRP (mg/L)	1.38 ± 0.07	1.28	1.91 ± 0.07	1.82	>0.05	1.45 ± 0.05	1.45	2.69 ± 0.16	2.61	<0.05	<0.05
H_2_O_2_ (*μ*mol/L)	12.70 ± 0.85	12.25	11.80 ± 0.81	11.07	>0.05	12.38 ± 0.90	11.91	16.0 ± 1.35	16.40	< 0.05	<0.05
NO (*μ*mol/L)	13.16 ± 0.46	13.55	13.57 ± 0.28	13.45	>0.05	13.14 ± 0.44	13.09	24.05 ± 0.47	24.35	<0.05	<0.05
3NT (nmol/L)	42.27 ± 2.91	41.53	52.91 ± 2.71	51.19	< 0.05	43.57 ± 2.54	42.64	34.69 ± 2.44	34.47	<0.05	<0.05
HSP27 (pg/mL)	736 ± 8.64	734	697 ± 15.45	696	>0.05	745 ± 12.95	747	814 ± 13.8	814	<0.05	<0.05

Abbreviations: TNF*α*: tumour necrosis factor *α*; IL-1*β*: interleukin 1*β*; hsCRP: high sensitivity C-reactive protein; H_2_O_2_: hydrogen peroxide; NO: nitric oxide; 3NT: 3-nitrotyrosine; HSP27: heat stress protein 27; SEM: standard error of the mean; Me: median.

**Table 3:** Lipoprotein-lipid profile before and after 6-day intermittent hypoxic exposure (mean ± SEM, Me).

	Control				1^st^ day vs. 6^th^ day	IHE				1^st^ day vs. 6^th^ day	Control vs. IHE 6^th^ day, *P* value
	1^st^ day, *n* = 6		6^th^ day, *n* = 6			1^st^ day, *n* = 6		6^th^ day, *n* = 6			
	x ± SEM	Me	x ± SEM	Me		x ± SEM	Me	x ± SEM	Me		
TG (mg/dL)	117 ± 17.07	109.5	131 ± 15.54	147	>0.05	109 ± 19.99	104.5	149 ± 15.55	156	>0.05	>0.05
TC (mg/dL)	179 ± 8.18	179	207 ± 5.09	203	>0.05	158 ± 13.94	162	157 ± 16.18	150	>0.05	**<**0.05
LDL (mg/dL)	111 ± 5.60	113	136 ± 5.32	130	>0.05	104 ± 9, 64	105	87 ± 16.33	84,7	>0.05	**<**0.05
ox-LDL **(**ng/mL)	4.73 ± 0.11	4.72	3.89 ± 0.15	3.93	<0.05	4.71 ± 0.12	4.65	3.28 ± 0.08	3.31	< 0.05	**<**0.05
HDL (mg/dL)	48 ± 1.81	47	44 ± 1.63	43	>0.05	43 ± 2.67	43,5	39 ± 1.57	39.5	>0.05	>0.05
Non-HDL (mg/dL)	131 ± 8.63	130.5	163 ± 5.40	130.5	>0.05	115 ± 16.2	116,5	118 ± 17.2	108	>0.05	**<**0.05

Abbreviations: TG: triglycerides; TC: total cholesterol; LDL: low-density lipoproteins; ox-LDL: oxidised low-density lipoproteins; HDL: high-density lipoproteins; non-HDL: cholesterol calculated by subtracting the HDL value from a TC; SEM: standard error of the mean; Me: median.

**Table 4:** Haematological variables before and after 6-day intermittent hypoxic exposure (mean ± SEM, Me).

	Control				1^st^ day vs. 6^th^ day	IHE				1^st^ day vs. 6^th^ day	Control vs. IHE 6^th^ day, *P* value
	1^st^ day, *n* = 6		6^th^ day, *n* = 6			1^st^ day, *n* = 6		6^th^ day, *n* = 6			
	*x* ± SEM	Me	*x* ± SEM	Me		*x* ± SEM	Me	*x* ± SEM	Me		
HB (g/dL)	14.9 ± 0.22	14.7	15.0 ± 0.32	14.7	>0.05	15.8 ± 0.21	15.7	14.4 ± 0.40	14.1	>0.05	>0.05
RBC **(**mln/mm^3^)	4.9 ± 0.12	4.9	4.99 ± 0.12	4.9	>0.05	5.1 ± 0.11	5.1	4.72 ± 0.14	4.6	>0.05	<0.05
RET (‰)	8.50 ± 0.61	8.5	7.33 ± 0.33	7.5	>0.05	9.00 ± 0.73	8.5	8.00 ± 0.36	8.0	>0.05	>0.05
HCT (%)	42.1 ± 0.67	41.9	44.4 ± 1.2	44.3	>0.05	44.9 ± 0.75	45.1	43.3 ± 1.33	42.3	>0.05	>0.05
MCV (fL)	85.5 ± 1.5	85.5	88.7 ± 1.3	89	>0.05	87.5 ± 0.71	87.0	91.7 ± 0.76	91.5	< 0.05	>0.05
MCH (pg/RBC)	30.2 ± 0.57	30	29.9 ± 0.20	29.7	>0.05	30.9 ± 0.43	30.9	30.6 ± 0.26	30.4	>0.05	>0.05
MCHC (g/dL)	35.3 ± 0.34	35.1	33.7 ± 0.30	33.3	<0.05	35.3 ± 0.26	35.4	33.3 ± 0.13	33.4	<0.05	>0.05
RDW (%)	12.7 ± 0.24	12.9	13.1 ± 0.10	13.0	>0.05	12.9 ± 0.24	13.0	12.8 ± 0.07	12.8	>0.05	<0.05

Abbreviations: HB: haemoglobin; RBC: red blood cells; RET: reticulocytes; HCT: haematocrit; MCV: mean cell volume; MCH: mean corpuscular haemoglobin; MCHC: mean corpuscular haemoglobin concentration; RDW: red cell distribution width; SEM: standard error of the mean; Me: median.

## Data Availability

The data used to support the findings of this study are available from the corresponding author upon request.
